# Regeneration pattern and genome-wide transcription profile of rhizome axillary buds after perennial rice harvest

**DOI:** 10.3389/fpls.2022.1071038

**Published:** 2022-11-28

**Authors:** Fan Yao, Qingyi Hu, Yingzhen Yu, Lifan Yang, Shuliang Jiao, Guangfu Huang, Shilai Zhang, Fengyi Hu, Liyu Huang

**Affiliations:** Key Laboratory of Biology and Germplasm Innovation of Perennial Rice from Ministry of Agriculture and Rural Affairs, School of Agriculture, Yunnan University, Kunming, Yunnan, China

**Keywords:** perennial rice, rhizome bud and axillary bud, regeneration regulation, transcriptome analysis, apical dominance

## Abstract

Perennial rice is a new type of rice that allows the harvest of rice for multiple years without growing new seedlings annually. This technology represents a green and sustainable agricultural production mode with many advantages for balancing agricultural ecology and food security. However, the differences in regeneration patterns between perennial and annual rice and the gene regulatory pathways of the apical dominance in axillary bud growth after harvest in perennial rice are still unclear. In this study, perennial rice (PR23) and annual rice (Chugeng28) were used to investigate axillary bud growth patterns before and after apical spike removal. After elimination of apical dominance at different development stages, perennial rice rhizome axillary buds at the compression nodes germinated more rapidly than others and developed into new seedlings. The axillary buds at the high-position nodes in annual rice grew faster than those at other nodes. Furthermore, the global gene expression patterns of PR23 rhizome buds at compression nodes grown for 1, 3, 4, and 5 days after apical spike removal were analyzed by transcriptome sequencing. Compared with the control buds without apical removal, 264, 3,484, 2,095, and 3,398 genes were up-regulated, and 674, 3,484, 1,594, and 1,824 genes were down-regulated in the buds grown for 1, 3, 4, and 5 days after apical spike removal, respectively. Trend analysis of the expressed genes at different time points was performed and co-expression network was constructed to identify key genes in rhizome axillary bud regrowth. The results showed that 85 hub genes involved in 12 co-regulatory networks were mainly enriched in the light system, photosynthesis-antenna protein, plant hormone signal transduction, ABC transporter and metabolic pathways, which suggested that hormone and photosynthetic signals might play important roles in the regulation of rhizome axillary bud regeneration in perennial rice. Overall, this study clarified the differences in the regeneration patterns of axillary buds between perennial and annual rice and provided insight into the complex regulatory networks during the regeneration of rhizome axillary buds in perennial rice.

## Introduction

Rice (*Oryza sativa* L.) is a staple food and feeds more than half of the world’s population (Tian et al., 2009; Lau et al., 2016). Traditional or annual rice production requires cultivation processes such as sowing seeds, raising seedlings, soil tillage, and transplanting in each planting season. Although people have mastered traditional planting techniques, they require large amounts of labor and fertilizer input. As society has developed, simplified, green and sustainable production modes have increasingly attracted the attention of rice breeders and producers. Compared with traditional annual rice, perennial rice production models can not only overcome cumbersome cultivation processes but also use less fertilizer and alleviate the soil erosion caused by continuous plowing, which brings significant economic, ecological, and social benefits (Huang et al., 2018). Perennial rice is a new type of rice cultivar selected for its rhizome trait (Hu et al., 2003; Zhang et al., 2017a; Zhang et al., 2017b). A series of perennial rice cultivars (lines), such as Perennial Rice 23 (PR23), Yunda25, Yunda101 and Yunda107, have been bred with genetic improvements by introgressing the rhizome traits of *Oryza longistaminata* into annual cultivated rice. PR23 is the world’s first certified perennial rice variety, and it became an important milestone for the study of perennial crops in 2018 (Zhang et al., 2017a; Huang et al., 2018; Zhang et al., 2019).

The rhizome axillary buds of perennial rice begin to elongate rapidly after grains are harvested, which seems to eliminate apical dominance. Then, new seedlings are formed for the next generation. Numerous studies have shown that auxin is the main regulator of plant apical dominance; it is synthesized at the shoot tip and transported down the stem and inhibits the growth of lateral buds (Snow, 1925; Skoog and Thimann, 1934; Umehara et al., 2008; Kieffer et al., 2010). Removal of the apex results in not only a reduction in auxin levels but also the loss of auxin polar transport (Morris and Johnson, 1990). Furthermore, plant hormones play important roles in controlling the branching of shoots, especially auxin and cytokinin (Liu et al., 2020), and the interactions of various hormones form multiple and complex regulatory pathways to achieve the developmental regulation of new meristems (Hu et al., 2011; Meitzel et al., 2021). The regeneration of plant axillary buds mainly involves cell division, tissue differentiation and organogenesis of the apical meristem (Kieffer et al., 2010; Su et al., 2011; Barbez et al., 2012).

The weakened development of lateral meristems is artificially selected during crop domestication for the pursuit of high grain yield (Wang and Li, 2005; Zhuo et al., 2020; Salam et al., 2021). Studying the regulatory mechanism of lateral meristem development after the removal of plant apical dominance is of great significance for finding the balance between the regeneration of rhizome axillary buds and grain yield in perennial rice.

In this study, we present evidence that axillary buds at different positions have different fates in both perennial and annual cultivated rice. Specifically, the rhizome axillary buds at the compression nodes become dominant in perennial rice after the elimination of apical dominance, while the axillary buds at the high-position nodes in annual rice grow faster than other nodes at different development stages. Moreover, key regulatory pathways and genes involved in hormone and photosynthetic signals contribute to the regulation of rhizome axillary bud regeneration in perennial rice. Our findings not only provide another vivid example of plant apical dominance but also demonstrate a new physiological mechanism by which photosynthetic signals might participate in the regulation of apical dominance.

## Materials and method

### Plant materials and phenotyping of axillary bud regeneration

PR23, a perennial rice cultivar independently bred by the Yunnan University and Yunnan Perennial Rice Technology Research Center, was released by the Yunnan Crop Committee in 2018. Chugeng28 is a locally popular *Geng* rice cultivar grown widely by farmers in Yunnan Province due to its high grain yield and exceptional grain quality. The sowing and transplanting dates of both PR23 and Chugeng28 in the first transplanting season were February 6 and March 15, 2021, respectively, in the Jinghong (Yunnan Province, China) experimental plot. In the growth seasons, the annual and perennial rice cultivars were managed according to local rice production.

Spike removal was performed at five developmental stages, flowering, filling, milk ripening, waxy ripening, and yellow ripeness, in both PR23 and Chugeng28. The lengths of the axillary buds at different positions were phenotyped before and 1, 3, 5, 7, 9, and 15 days after apical spike removal. At each time point, 10 rice plants (tillers n > 40) were investigated for regeneration pattern analysis of axillary buds. Experimental data were analyzed with the IBM SPSS statistical package v.20.0 (SPSS, Inc., Chicago, IL, USA), and the figures were generated using Origin 2015 (Sys Software, Inc.). One-way analysis of variance (ANOVA) was implemented to compute differences within groups. A level of statistical significance of *p * <  0.05 was set. SPSS software was used to analyze the variation in axillary bud length at different positions at 15 days after apical removal *via* Duncan’s multiple comparison.

### Sampling of axillary buds and RNA-sequencing

After PR23 entered the yellow ripeness stage, transcriptome sampling was performed at 0, 1, 3, 4, and 5 days after harvest. The samples were named T0d, T1d, T3d, T4d, and T5d. After the PR23 harvest, the stubble height was approximately 5-10 cm above the ground. Fifteen axillary buds at the compression nodes were taken as biological replicates, and three biological replicates were taken at each time point. Samples were wrapped in tin foil, quickly placed in liquid nitrogen, and then stored at -80°C.

The total RNA of each sample was extracted by TRIzol^®^ (Invitrogen Company), the RNA integrity was analyzed by RNase agarose gel electrophoresis and the Bioanalyzer 2100 system, and the RNA purity was detected by the NanoPhotometer^®^ spectrophotometer. mRNA was enriched by Oligo(dT) beads and then digested and reverse-transcribed with random primers and RNase H to synthesize first-strand cDNA. This was followed by the addition of buffer, dNTPs, and DNA polymerase I to synthesize double-stranded cDNA, which was eluted with QiaQuick PCR kits and EB buffer, and then subjected to end repair. A TruSeq RNA Sample Preparation Kit (Illumina) was used to perform paired-end sequencing on qualified samples, and complete library construction sequencing was performed on the Illumina NovaSeq 6000 platform by Beijing Novegene Co., Ltd. The raw sequence reads were submitted to the Genome Sequence Archive (GSA) in the National Genomics Data Center (NGDC) under the accession number CRA007317.

### Bioinformatics analysis

Low-quality nucleotides (< Q20) were trimmed from the raw sequences for each sample, and then paired-ended reads with lengths of less than 30 bp were removed using an in-house Perl script. Using HISAT2 software, clean reads were aligned to the *O. sativa* cv. Nipponbare IRGSP 1.0 reference genome (Kawahara et al., 2013; Pertea et al., 2016). Differentially expressed genes (DEGs) of T1d, T3d, T4d, and T5d compared with T0d were screened according to the FPKM (Fragments Per Kilobase of transcript per Million mapped reads) value, which was calculated to quantify the gene expression level by featureCounts software (Liao et al., 2014). Genes/transcripts with a false discovery rate (FDR) below 0.05 and | log_2_ (fold change) | ≥ 1 were considered differentially expressed genes/transcripts by DESeq2 software (Love et al., 2014). Gene Ontology (GO) (Ashburner et al., 2000) and Kyoto Encyclopedia of Genes and Genomes (KEGG) (Kanehisa and Goto, 2000) analyses were performed as conventional methods. All calculated *p* values were subjected to FDR correction, taking FDR ≤ 0.05 as a threshold.

Weighted gene co-expression network analysis (WGCNA) was performed on the OmicShare cloud platform (https://www.omicshare.com/tools) with R language 3.3.2 using the WGCNA package (Langfelder and Horvath, 2008). A co-expression module with high similarity (> 75%) and a gene clustering tree were constructed according to the correlations between the expression levels of the genes (Du et al., 2017). Cytoscape 3.3.0 was used to display the co-regulation network (Lopes et al., 2010).

### Quantitative real-time reverse transcription-PCR

Gene sequences were obtained from the Nipponbare reference sequence (Kawahara et al., 2013). Primer 5.0 software was used to design primers, and all primers, including those for the internal reference gene *Actin1*, were designed according to the sequence of the selected genes (**Supplementary Table 1**). The total RNA of each sample was extracted with the Hipure Plant RNA Mini Kit and reverse transcribed into cDNA, and the product was used as a template for qRT-PCR. Each reaction was set up with 3 biological replicates, and the *PerfectStart* ^®^ Green qPCR SuperMix kit was used to carry out qRT-PCR and 2^-ΔΔCT^ analyses of the relative transcript levels for each gene (Bustin et al., 2009).

## Results

### Regeneration patterns of axillary buds in perennial and annual rice

**Figure 1:**
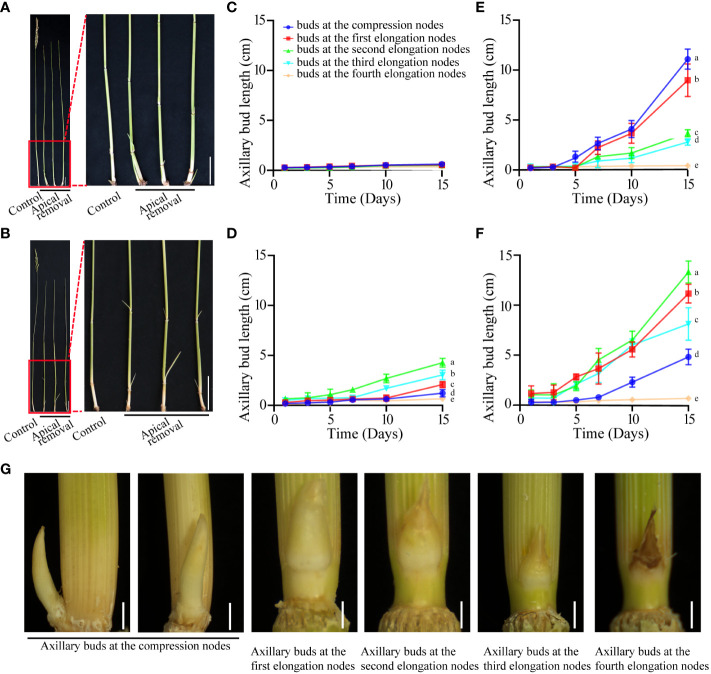
Regeneration patterns of axillary buds in perennial and annual rice before and after apical spike removal. Phenotype of axillary buds of PR23 **(A)** and Chugeng28 **(B)** at 5 days after apical spike removal. Control indicates intact plants. Scale bars: 10 cm. Growth dynamics of axillary buds in PR23 **(C)** and Chugeng28 **(D)** plants without apical spike removal. Growth dynamics of axillary buds in PR23 **(E)** and Chugeng28 **(F)** with apical spike removal. Data in **(B, C, E, F)** are means ± SDs (n > 40). Different letters within a column represent significant differences at *p* < 0.05. Surveys were performed at 1, 3, 5, 7, 10, and 15 days after apical spike removal. **(G)** Phenotype of axillary buds in different nodes of PR23 after 5 days of apical spike removal at the yellow ripeness stage. Scale bars: 0.5 mm.

The axillary buds of both PR23 and Chugeng28 have similar performance in the intact plant at the mature stage. Before harvesting, the axillary buds at each node showed inhibited growth (**Figures 1A-D**). The spikes were removed when PR23 and Chugeng28 entered the yellow ripeness stage. Dynamic measurement of the regeneration of axillary buds indicated that the rhizome buds at the compression nodes in PR23 germinated fastest and then grew out from the soil to become seedlings (**Figure 1E**). The axillary buds at the high-position nodes in Chugeng28 grew faster than those at the other nodes (**Figure 1F**). In other words, all the axillary buds of PR23 and Chugeng28 may be inhibited by apical dominance. Regeneration differences in axillary buds at different positions were obvious at 5, 7, 10, and 15 days after apical removal (**Figures 1E, F**). Moreover, the rhizome buds at the compression nodes have the potential to form independent seedlings. With the growth of rhizome buds at compression nodes, buds at other positions grew slowly and even gradually tended to die (**Figure 1G**).

Almost all *Oryza* lines have the potential to regenerate new panicles from axillary buds before the plant becomes senescent (Wang et al., 2020). To determine whether perennial and annual rice cultivars are different in regenerating new panicles from axillary buds before the plant becomes senescent, we surveyed the regeneration patterns of axillary buds in PR23 and Chugeng28 at three developmental stages: flowering, filling, milk ripening and waxy ripening. Interestingly, we found that the rhizome buds at the compression nodes in PR23 were always dominant compared with others after apical spike removal at all stages. Chugeng28 has the potential to regenerate from axillary buds at high positions at all stages (**Supplementary Figure 1**). Perennial and annual rice established different dominant axillary buds after apical spike removal, which resulted in two kinds of plant architecture in the next growth cycle.

### Global gene expression profiling in dominant rhizome buds of PR23 before and after apical spike removal by transcriptome sequencing

**Figure 2:**
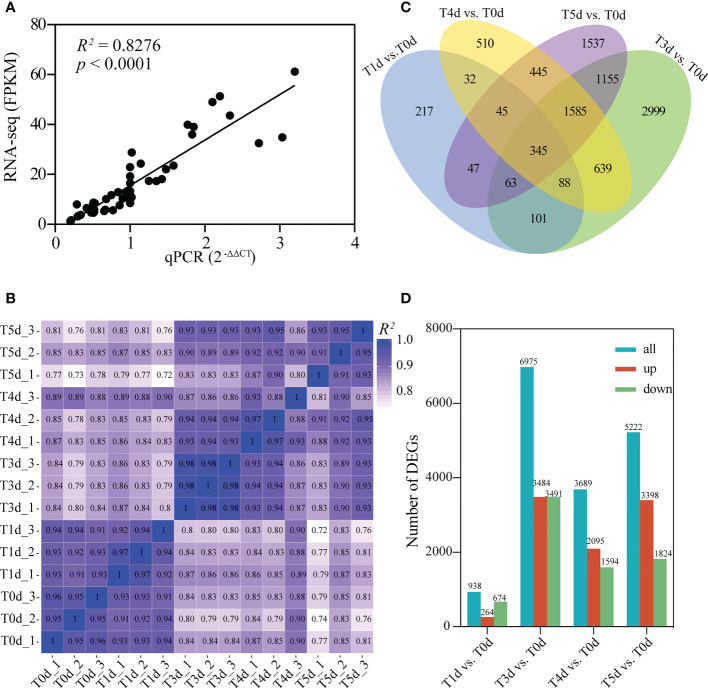
Transcriptome data analysis of regenerating rhizome buds of compression nodes in PR23. **(A)** Comparison of transcription measurements by Illumina sequencing and qRT-PCR assays. The correlation coefficient (*R^2^*) between the two datasets was 0.83. **(B)** Pearson correlation between samples. **(C)** Venn diagram of DEGs at T1d, Td3, T4d, and T5d compared with T0d, respectively. **(D)** Numbers of DEGs are showed in blue bars in each comparison, including up-regulated (shown in red bars) and down-regulated DEGs (shown in green bars).

To determine the gene expression patterns of dominant buds after apical spike removal in perennial rice, rhizome buds at the compression nodes in PR23 at 0, 1, 3, 4, and 5 days after spike removal were analyzed by transcriptome sequencing. The clean reads of each sequencing sample were between 39,332,930 and 44,350,522, and the clean bases of the original filtered data were between 5.90 G and 6.65 G. There were between 39,981,220 and 45,104,876 raw reads, and the error rates were between 0.02% and 0.03% across the 15 sequencing samples. After filtration, the highest proportion of bases with mass values greater than Q20 was 98.07%, and the lowest value was 97.42%. The proportions of bases with mass values greater than Q30 were between 93.03% and 94.52%, and the average GC contents were between 52.86% and 54.4%, indicating that the base recognition was high and the quality was reliable (**Supplementary Table 2**). A total of 38,759 genes were obtained from the above sequenced samples. To verify the reliability of the sequencing data, we used qRT-PCR to analyze the expression levels of the transcriptome sequencing results. The correlation coefficient *R^2^* = 0.83 (**Figure 2A**) and Pearson correlation analysis (**Figure 2B**) indicated that the transcriptome sequencing data were reliable and could be used for further analysis. Differential expression analysis showed that compared with the control sample (T0d) before apical spike removal, 938, 6,975, 3,689, and 5,222 differentially expressed genes (DEGs) were obtained at 1, 3, 4, and 5 days after apical spike removal in PR23, respectively (**Figures 2C, D** and **Supplementary Table 3**). The number of DEGs in T3d vs. T0d was much greater than those at other time points, which indicates that apical dominance signal elimination requires more than 24 hours to fully activate the growth of rhizome buds, and the apical meristem of the rhizome buds became active at 3 days after apical spike removal. Then, the rhizome buds rapidly grew and elongated, which is in accordance with the above phenotypic observation (**Figure 1G**) and the results of Pearson correlation analysis (**Figure 2B**).

### Pathway enrichment of DEGs at T1d, T3d, T4d, and T5d vs. T0d

To determine the internal response or regulation pathway of rhizome buds after harvest in perennial rice, GO and KEGG enrichment analyses were performed to classify DEGs at different time points. Interestingly, except for DEGs in T1d vs. T0d, genes of enriched GO categories and KEGG terms were mostly up-regulated after apical removal (**Supplementary Tables 4**, **5**).

Compared with T0d, 49 categories were significantly enriched in the T1d group. Ribosome-related pathways were annotated as being the most enriched for up-regulated genes. Metabolic processes were annotated as being the most enriched for down-regulated genes (**Supplementary Table 4**). An approximate result was also obtained in KEGG analysis, including 52 up-regulated genes in the ribosome (ko03010) pathway and many down-regulated genes in carotenoid biosynthesis (osa00906), flavonoid biosynthesis (osa00941), starch and sucrose metabolism (osa00500), and phenylpropanoid biosynthesis (osa00940) (**Supplementary Table 5**).

There were 128 significantly enriched categories in T3d vs. T0d, which was much more than the number of pathways in T1d. In addition to ribosome-related pathways, photosynthesis-related pathways and cell division-related pathways were also significantly enriched among the up-regulated genes. Moreover, almost all ADP binding (GO: 0043531)-related genes enriched were down-regulated (**Supplementary Table 4**). The KEGG terms, ribosome (osa03010), photosynthesis (osa00195), photosynthesis-antenna proteins (osa00196), phenylpropanoid biosynthesis (osa00940), oxidative phosphorylation (osa00190) and starch and sucrose metabolism (osa00500), were significantly enriched, and most genes in these pathways were up-regulated (**Supplementary Table 5**). In the T4d vs. T0d comparison group, 87 GO categories were significantly enriched. Among the significantly enriched terms, ribosome-related pathways, photosynthesis-related pathways, and cell division-related pathways overlapped with GO and KEGG classifications enriched in T3d (**Supplementary Tables 4**, **5**).

In the comparison group of T5d vs. T0d, 66, 44, and 52 GO categories were significantly enriched in the biological process, cell composition and molecular function categories, respectively (**Supplementary Table 4**). According to DEGs at different time points, more pathways enriched in the GO and KEGG classifications were enriched in T5d. GO categories included photosynthetic-, cell division-, and hydrolase activity-related pathways, and KEGG pathways included photosynthesis, phenylpropanoid biosynthesis, amino sugar and nucleotide sugar metabolism, indicating that rapid growth and development are taking place in T5d (**Supplementary Tables 4**, **5**).

Among the four comparison groups, 345 DEGs were commonly regulated at different time points after apical spike removal (**Figure 2C**). GO and KEGG analyses of these DEGs indicated that metabolism-related pathways were rapidly regulated and lasted for a few days after harvest (**Supplementary Figure 3**). Cell division- and photosynthesis-related pathways were rapidly induced at 3 days after apical spike removal in PR23 (**Supplementary Tables 4**, **5**).

### Co-regulatory networks and core genes regulating the regeneration of dominant rhizome buds in PR23

**Figure 3:**
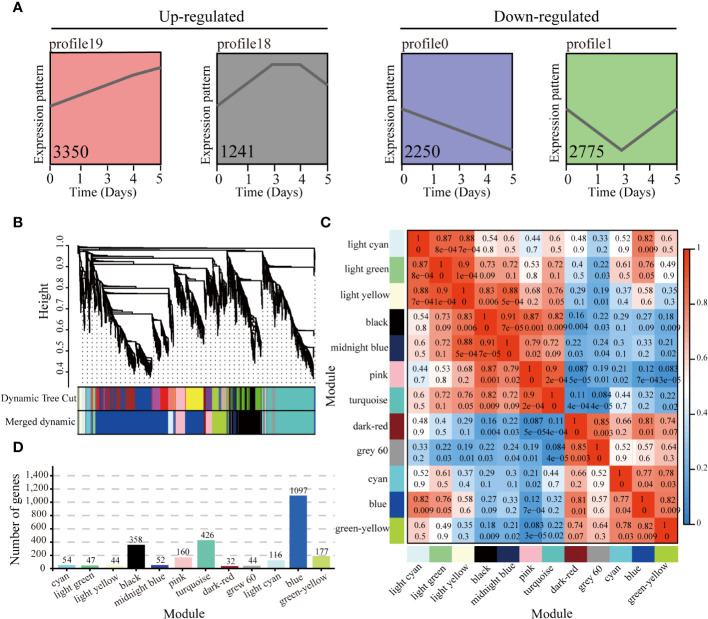
WGCNA of expressed genes in significant expression trends of profiles 0, 1, 18, and 19. **(A)** Significant expression trends of profiles 19, 18, 0, and 1 (with *p* ≤  0.05) at 0, 1, 3, 4, and 5 days after apical spike removal. The gene number containing in each expression profile is listed in the profile. **(B)** Hierarchical cluster tree showing co-expression modules identified by WGCNA. Each leaf in the tree represents one gene. **(C)** Pairwise correlation coefficients between modules. Rows and columns are the module names, and numbers represent coefficient values and *p* values. **(D)** The number of genes corresponding to each module.

The gene expression trends of 38,759 genes expressed at different time points after harvest in PR23 were analyzed, and a total of 20 trend profiles were obtained (**Supplementary Figure 2**). According to the test of significance for the gene expression trend analysis (*p* < 0.05), and consistent with the continuous sampling of axillary buds, four target trend profiles, including continually increasing expression (profile 19), expression first increasing and then decreasing (profile 18), steadily decreasing expression (profile 0), and expression first decreasing and then increasing (profile 1), were selected for WGCNA, among which 3,350, 1,241, 2,250, and 2,775 genes were in profiles 19, 18, 0, and 1, respectively (**Figure 3A** and **Supplementary Figure 2**).

A total of 9,616 genes in profiles 19, 18, 0 and 1 were analyzed to identify the co-expression network and core genes contributing to PR23 rhizome bud regeneration. According to the dynamic shear method of system clustering, the modules with large similarity (> 75%) were merged, and 12 co-expression modules were obtained (**Figures 3A–C**). The modules with different colors represent different expression patterns. The blue module had the largest number of genes, 1,097, and the dark red module had the smallest number of genes, 32 (**Figure 3D**). The expression patterns of the blue, cyan, green−yellow, dark red, light cyan, and gray 60 modules were up-regulated, and the expression patterns of the pink, black, midnight blue, light yellow, turquoise and light green modules were down-regulated (**Figure 3**; **Supplementary Figure 2**, and **Supplementary Table 6**).

**Figure 4:**
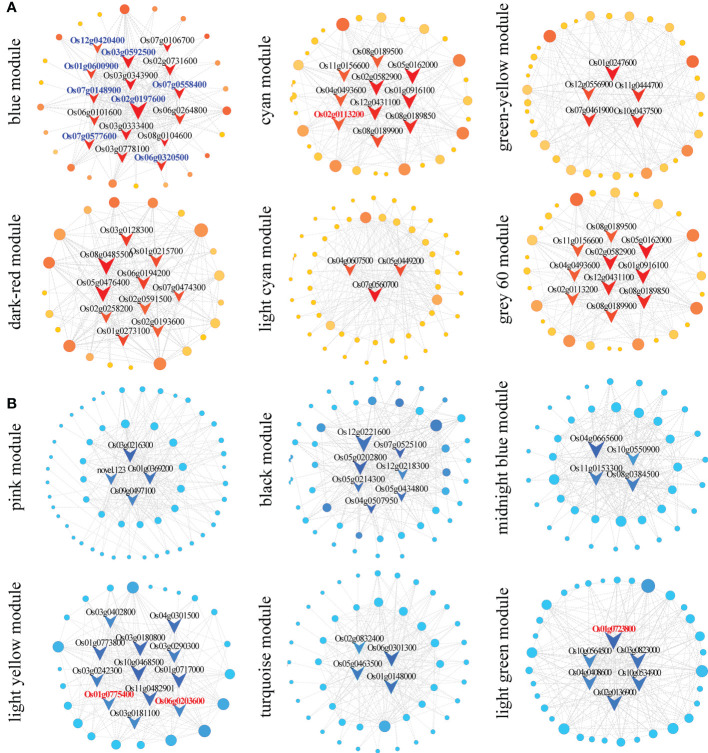
Co-expression network construction and hub gene screening. **(A)** Construction of the co-expression network of up-regulated modules (blue, cyan, green-yellow, dark red, light cyan, and gray 60 modules). **(B)** Construction of the co-expression network of down-regulated modules (pink, black, midnight blue, light yellow, turquoise, and light green modules). The nodes in the figure represent identified DEGs, the connecting lines between genes represent co-expression correlations, and the red and dark blue V-shaped nodes represent core or hub genes. Gene ID in blue and red color represent photosynthesis- and phytohormones-related genes, respectively.

Furthermore, we constructed a co-regulatory network of genes with module eigenvalues > 0.75 in the above 12 target modules (**Figure 4**). A total of 85 core/hub genes were identified. Among the up-regulated patterns, there were 16, 10, 5, 10, 3 and 4 hub genes in the blue, cyan, green−yellow, dark red, light cyan, and gray 60 modules, respectively (**Figure 4A** and **Supplementary Table 6**). Among the down-regulated patterns, there were 4, 7, 4, 12, 4, and 6 core genes in the pink, black, midnight blue, light yellow, turquoise, and light green modules, respectively (**Figure 4B** and **Supplementary Table 6**).

Apical dominance directly or indirectly inhibits the growth of axillary buds due to the polar transport of auxin formed at the apex during plant growth and development (Cao and Jiao, 2020). The regeneration of axillary buds is an important determinant of crop yield, and the elongation of axillary buds in ratoon rice is regulated by phytohormones (Xu et al., 2020). In this study, one auxin-related transcription factor, *OsIAA12* (*Os03g0633800*), and a peroxidase (*Os10g0109300*) were identified in the co-regulatory network of the blue module (**Supplementary Table 6**).

The content of flavonoids is related to the polar transport of auxin. In the light yellow module, whose expression trend was profile 1, there were also two enzymes related to flavonoid content: chalcone isomerase (*Os06g0203600*) and 4-coumaric acid: coenzyme A ligase (*Os08g0448000*). In the light green module, there were ABC transporters related to hormone transport (*Os05g0384600* and *Os01g0723800*) (**Supplementary Table 6**).

Three hub genes (*Os02g0113200*, *Os09g0441400* and *Os01g0775400*), one each in the cyan, light green and light yellow modules, function in cytokinin synthesis. *Os09g0441400* and *Os02g0113200* are functionally annotated as cytochrome P450, while *Os01g0775400* is a cytokinin dehydrogenase 5 precursor (**Supplementary Table 6**).

In addition to plant hormone-related genes, 17 genes related to photosynthesis were found in the blue module, which showed up-regulated expression trends at all time points. These genes included six optical system I-related proteins (*Os08g0560900*, *Os09g0481200*, *Os07g0148900*, *Os12g0420400*, *Os03g0778100*, and *Os04g0414700*), three optical system II-related proteins (*Os03g0333400*, *Os07g0544800*, and *Os01g0501800*), six optical antenna proteins (*Os01g0600900*, *Os06g0320500*, *Os07g0558400*, *Os02g0197600*, *Os03g0592500*, and *Os07g0577600*), one ferrite-reducing protein related to photosynthetic electron transport (*Os08g0104600*), and one chloroplast ATP synthase (*Os07g0513000*) (**Supplementary Table 6**).

## Discussion

### Different fates of regenerated axillary buds in perennial and annual rice

Apical dominance is a growth regulation mechanism that directly or indirectly inhibits the growth of axillary buds during plant growth and development (Cao and Jiao, 2020). It is generally believed that the axillary buds of all rice varieties have regeneration potential after apical spike removal before maturity, but the regeneration patterns are different. Perennial rice is a type of multiharvest rice that utilizes the characteristics of vegetative propagation found in *O. longistaminata* (Hu et al., 2011), in which new tillers that emerge from the rhizome are maintained for successive reproduction. The regeneration of high-position axillary buds into panicles for a second cycle of reproduction in ratoon rice is regulated by phytohormones (Xu et al., 2020). The results also showed that the effective removal of apical dominance would greatly promote the growth of axillary buds in both perennial and annual rice, but these rice types are different in their determinants of dominant axillary buds. Annual rice showed rapid growth of high-position axillary buds after apical spike removal in different growth periods, while perennial rice showed rapid germination of rhizome buds at the compression nodes. We preliminarily believe that the determinants of dominant axillary buds in perennial rice and annual rice are not dependent on apical removal. Treatment of apical dominance removal can promote rhizome buds to grow rapidly and then form a seedling or tiller in perennial rice, as well as a panicle in annual rice.

Rhizomes determine the vegetative propagation of *O. longistaminata*, which always persists the juvenile stage (Fan et al., 2020). Tillers that emerged from these rhizomes, maintained in the juvenile stage were similar to seedling plants with their own roots. Although we do not know if compression nodes in PR23 are exactly the same as the rhizomes of *O. longistaminata* due to their different morphological characteristics, tillers regenerated from compression nodes in perennial rice formed from the hybridization of *O. sativa* and *O. longistaminata* have similarities with seedling plants. After the ripening stage, the rice panicle became senescent and died, and rhizome buds at the compression nodes entered a new growth cycle. This phenomenon was also discovered in rose, in which branches die after flowering and new shoots bloom again (Li et al., 2015). All the results further clarified the effect of apical dominance on the development of the shoot apical meristem (SAM), which is beneficial to improve the yield of perennial rice in actual production.

### In addition to phytohormones, the photosynthetic pathway plays important roles in rhizome bud regeneration in perennial rice

Numerous studies have shown that phytohormones play key roles in apical dominance and axillary bud regeneration (Liu et al., 2012); for example, the polar transport of auxin synthesized at the apex inhibits the differentiation and regeneration of axillary buds (Guo et al., 2020), and *in situ* cytokinin synthesis promotes the differentiation of meristems and the regeneration of axillary buds (Mueller and Leyser, 2011). Hormone signals coordinate with environmental signals to form a complex regulatory network to jointly regulate the initiation and elongation of plant buds (Serre et al., 2021; Patil et al., 2022). In our study, auxin synthesis- and transport-related genes, including one Aux/IAA-related gene (*OsIAA12*, *Os03g0633800*) and two flavonoid synthase-related genes (4-coumarate coenzyme A ligase, *Os4CL5*, *Os08g0448000* and chalcone isomerase, *Os06g0203600*), were identified to play crucial roles in the regeneration of rhizome buds in PR23. The decreased expression of *Os4CL5* and chalcone isomerase *Os06g0203600* in the rhizome buds of compression nodes after removing the apex in perennial rice led to a decrease in flavonoid content. These variations are consistent with the harvest of perennial rice production. Afterward, a decrease in the flavonoid content results in reduced polar transport of auxin after the removal of spikes in PR23. With the reduction of auxin in axillary buds, the dormancy of the SAM is broken. The SAM in the elongated axillary bud becomes apical and builds a new apical dominance system (Wai and An, 2017). In our study, a few days later, the expression levels of *Os4CL5* and chalcone isomerase genes gradually increased to regulate the polar transport of auxin and reduce the concentration of auxin at the SAM of the rhizome buds. The continuous increase in *OsIAA12* expression led to the enhanced perception of a low auxin signal in the meristem, which further promoted the growth of tillers.

The expression trends of three cytokinin-related genes (*Os09g0441400*, *Os02g0113200* and *Os01g0775400*) were consistent with those of *Os4CL5* and chalcone isomerase *Os06g0203600*, which further suggested that cytokinin played a regulatory role in rhizome axillary bud regeneration. Cytokinin dehydrogenase (*Os01g0775400*) is a rate-limiting enzyme in the cytokinin degradation pathway. The understanding of cross-signaling between different hormones is not profound, and the complex regulatory network formed among different signals needs further study.

Nutrient metabolism and energy transfer play important roles in the regeneration of axillary buds (Evers et al., 2006; Girault et al., 2008). Seventeen genes in photosynthetic pathways covering the light-harvesting antenna complex and the electron transport chains (linear and ring electron transport chains) were unexpectedly induced in rhizome buds after harvest, providing a new viewpoint into the regeneration of rhizome axillary buds. It is easy to imagine that the overall photosynthetic capacity of the plant decreases dramatically after harvest, which removes many photosynthetic organs (especially the leaves). Therefore, we speculate that the remaining tissue was induced to promote photosynthesis to compensate for the deficient energy in rhizome axillary buds. Although the results of co-regulation network analysis need to be further verified, the co-expression network provides a favorable theoretical basis to analyze the rhizome axillary bud regeneration mechanism of perennial rice.

### Summary and future research prospects

Compared with annual rice, we altered the dominant axillary buds of plant with the introgression of perenniality from *O. longistaminata*. This distant hybridization breeding solved the problem of energy balance between grain yield and the regeneration of axillary or rhizome buds. Moreover, these novel types of rice, perennial rice, have different regulation pathways after harvest and can generate new seedlings for next-generation production. In actual production, after the first harvest of perennial rice, the germination of regenerated rhizome buds was inconsistent, which made field management difficult and seriously affected the continuous high and stable yield of perennial rice. Further studies are needed to overcome this problem by refining breeding and cultivation techniques, such as regeneration regulation by exogenous hormone treatment.

## Data availability statement

The data presented in the study are deposited in the Genome Sequence Archive (GSA) in the National Genomics Data Center (NGDC) repository, accession number CRA007317.

## Author contributions

LH and FH designed the experiments and wrote the manuscript. FY, QH, and YY performed the experiments. FY, QH, YY, LY and SJ performed the phenotyping analysis. GH and SZ gave advice for this program. All authors contributed to the article and approved the submitted version.

## Funding

This work was supported by grants from the Yunnan Provincial Science and Technology Department (202001BB050050, 202005AC160001, and 202005AF150009) and Graduate Innovation Project (KC-22222593) of Yunnan University.

## Conflict of interest

The authors declare that the research was conducted in the absence of any commercial or financial relationships that could be construed as a potential conflict of interest.

## Publisher’s note

All claims expressed in this article are solely those of the authors and do not necessarily represent those of their affiliated organizations, or those of the publisher, the editors and the reviewers. Any product that may be evaluated in this article, or claim that may be made by its manufacturer, is not guaranteed or endorsed by the publisher.
