# Supplementary material for: Regeneration pattern and genome-wide transcription profile of rhizome axillary buds after perennial rice harvest
Source: Front Plant Sci. 2022 Nov 28;13:1071038. doi: 10.3389/fpls.2022.1071038 (PMC9742242; doi:10.3389/fpls.2022.1071038)
Supplement: Supplementary file 1 [file DataSheet_1.docx]

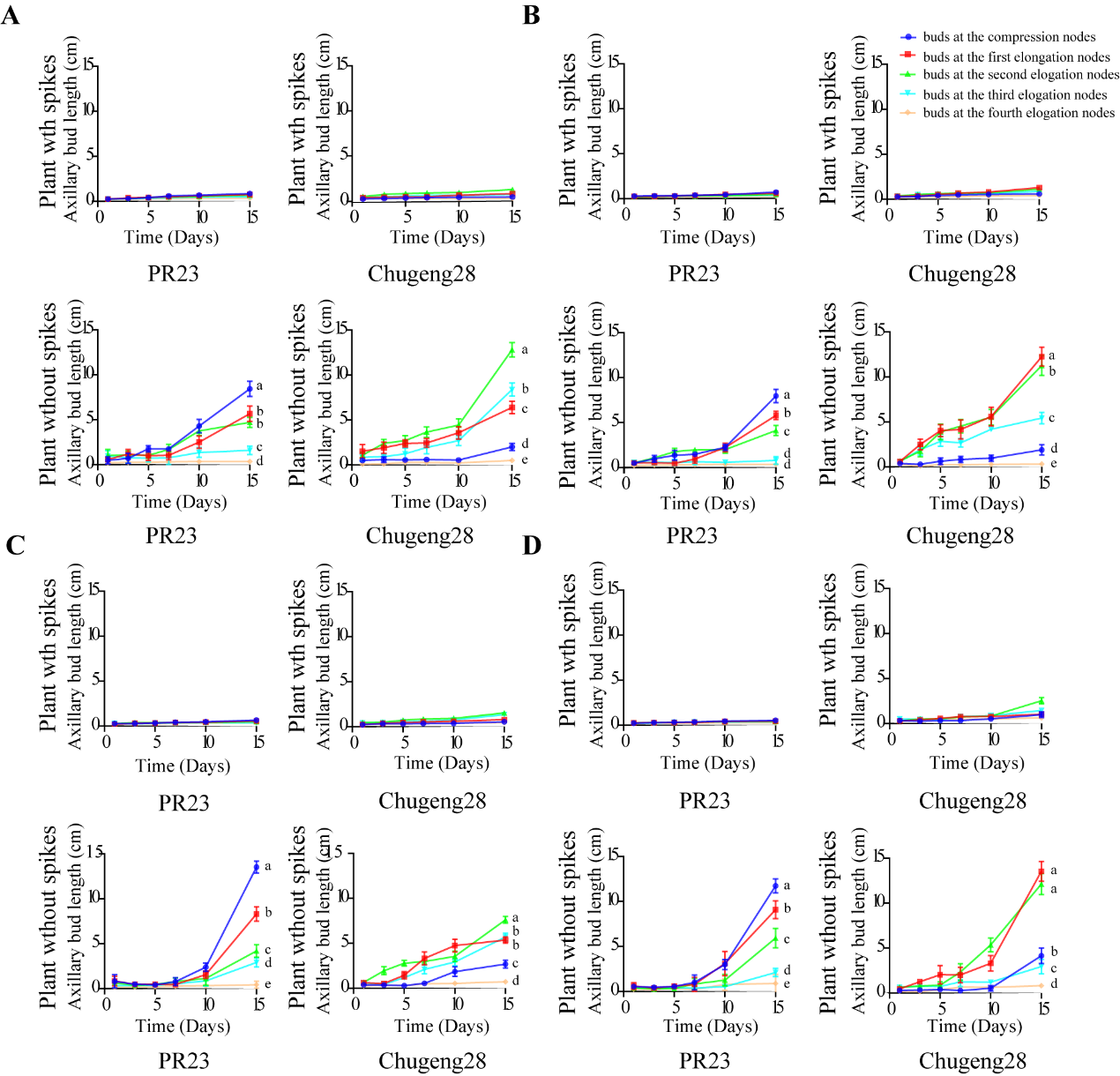


**Supplementary Figure 1** Growth dynamics of axillary buds in PR23 and Chugeng28 plants before and after apical spike removal at the flowering (A), filling (B), milk ripening (C), and waxy ripening (D) developmental stages. Surveys were performed at 1, 3, 5, 7, 10, and 15 days after apical spike removal. Values are expressed as the means ± SDs, n > 40. Significantly different values (*p* < 0.05) are indicated by different letters.


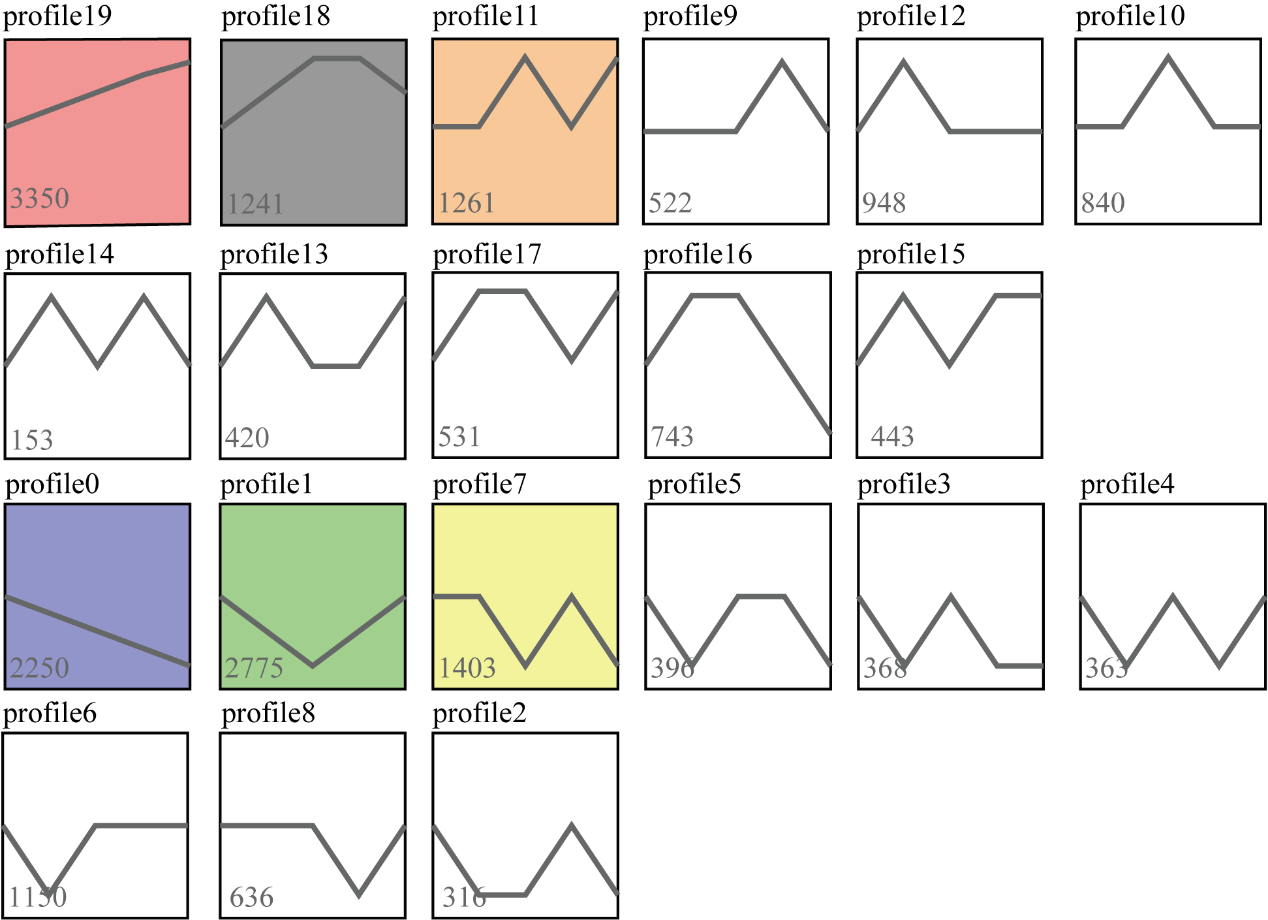


**Supplementary Figure 2** Trend analysis of all expressed genes at 0, 1, 3, 4, and 5 days after spike removal. Block diagrams with colors indicate trend profiles with a significance level under the cutoff for statistical analysis (*p* < 0.05). The numbers in the blocks indicate counts of genes in the trend profiles.


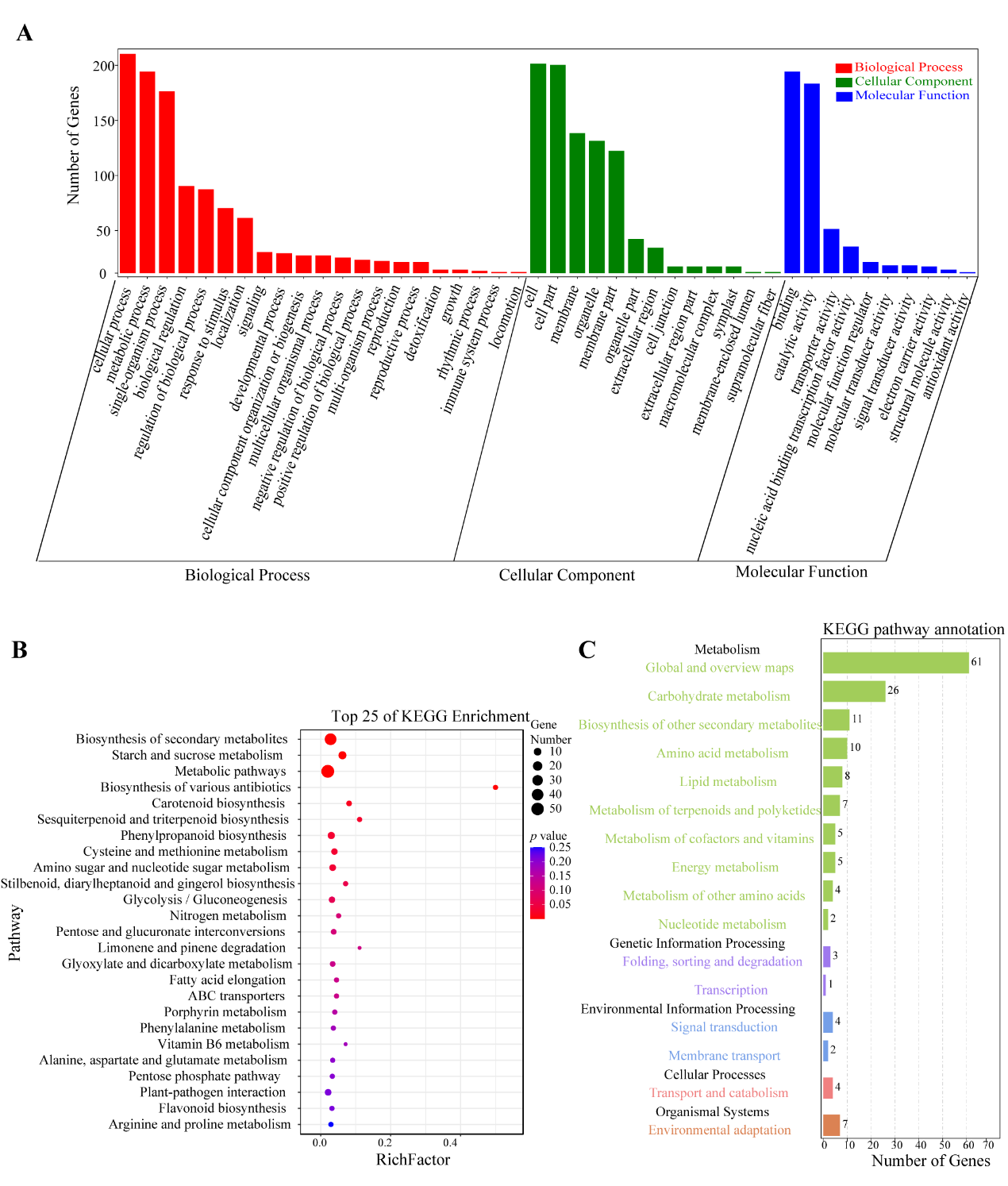


**Supplementary Figure 3** GO and KEGG enrichment of the common DEGs at different time points after apical spike removal. **(A)** DEGs were enriched in three GO database classifications, including molecular function, biological process, and cellular composition. **(B)** Bubble diagram of the top 25 pathways enriched by KEGG analysis. **(C)** Bar chart of KEGG annotation secondary classification.
