# Supplementary material for: Regeneration pattern and genome-wide transcription profile of rhizome axillary buds after perennial rice harvest
Source: Front Plant Sci. 2022 Nov 28;13:1071038. doi: 10.3389/fpls.2022.1071038 (PMC9742242; doi:10.3389/fpls.2022.1071038)
Supplement: Supplementary file 2 [file Table_1.docx]

**Supplementary Table 1 Information on the primers used in qRT-PCR analysis.**

| **Gene ID** | **Forward Primer (5'-3')** | **Reverse Primer (5'-3')** |
| --- | --- | --- |
| LOC_Os03g50890 (*Actin1*) | GCTCCGTGGCGGTATCAT | CGGCAGTTGACAGCCCTAG |
| LOC_Os07g35510 | GACTACTACCAACGCAAGGGC | CGATTTCGCAACGCACC |
| LOC_Os06g06050 | GGAACACAACCGAACAACCCT | CAGCAGAAATGAGCGGAGAGAG |
| LOC_Os01g47070 | TCCCCTCTCCTTGCCCTCTT | GAAGGTGCTGAGGAAGGCGA |
| LOC_Os02g41550 | CGTGGTTTGGTTCAGGAGG | GAAGACAGGGAGCACAGCG |
| LOC_Os01g71340 | CATTGGTCCTTGGAGTTGC | GTGAGGGCGATGCTTGAG |
| LOC_Os03g17350 | TCCACTACATCTCGCTCGTCA | TCCAGAGGCACTCCCACTTG |
| LOC_Os04g58710 | CGATCATGGAGAAGCTGGAG | CGCCATCTCCTGACCCAC |
| LOC_Os11g29840 | TGACAAAGAAGAGGGCGAAGC | GAGGTGACGATTGAGCGACG |
| LOC_Os09g29404 | AACTGAAGATGAGAAAGGTGC | CCCAATCAAAAGGAGAGC |
| LOC_Os12g43660 | CAAGTCCTCCAACATCCTCCT | AGCGAGGTAGCCGTAGGTG |
| LOC_Os08g07010 | GCCGCCAACTCTTACCCTCT | CTCCTTCGCCACTGCCG |
| LOC_Os05g32710 | AAGGCTAAGGCAGATACGCAA | CATCCCAACCTGGCTCACAT |
| LOC_Os06g50340 | TGCTGGCTCCTACGGCTACAT | GCTCCAATAGCACCACACCG |
