# Supplementary material for: Regeneration pattern and genome-wide transcription profile of rhizome axillary buds after perennial rice harvest
Source: Front Plant Sci. 2022 Nov 28;13:1071038. doi: 10.3389/fpls.2022.1071038 (PMC9742242; doi:10.3389/fpls.2022.1071038)
Supplement: Supplementary file 3 [file Table_2.docx]

**Supplementary Table 2 Filtered sequencing sample data.**

| **Sample** | **Raw-reads** | **Clean-reads** | **Clean-bases** | **Error-rate (%)** | **Q20 (%)** | **Q30 (%)** | **GC-pct (%)** |
| --- | --- | --- | --- | --- | --- | --- | --- |
| T0d-1 | 41,846,954 | 41,239,920 | 6.19G | 0.02 | 97.95 | 94.25 | 53.30 |
| T0d-2 | 41,243,292 | 40,654,056 | 6.10G | 0.02 | 98.03 | 94.42 | 53.02 |
| T0d-3 | 43,463,960 | 42,845,736 | 6.43G | 0.03 | 97.93 | 94.18 | 53.58 |
| T1d-1 | 45,104,876 | 44,350,522 | 6.65G | 0.02 | 98.07 | 94.52 | 53.45 |
| T1d-2 | 41,472,020 | 40,825,794 | 6.12G | 0.02 | 97.98 | 94.22 | 53.17 |
| T1d-3 | 43,004,816 | 42,313,310 | 6.35G | 0.03 | 97.97 | 94.18 | 52.86 |
| T3d-1 | 44,212,142 | 43,693,466 | 6.55G | 0.02 | 97.99 | 94.31 | 53.10 |
| T3d-2 | 43,863,628 | 43,185,224 | 6.48G | 0.02 | 97.97 | 94.25 | 53.3 |
| T3d-3 | 42,145,640 | 41,526,688 | 6.23G | 0.02 | 98.03 | 94.41 | 53.19 |
| T4d-1 | 42,416,044 | 41,987,420 | 6.30G | 0.03 | 97.42 | 93.03 | 54.27 |
| T4d-2 | 39,981,220 | 39,332,930 | 5.90G | 0.02 | 97.99 | 94.28 | 53.58 |
| T4d-3 | 42,625,962 | 42,046,270 | 6.31G | 0.02 | 98.01 | 94.41 | 53.57 |
| T5d-1 | 41,864,734 | 41,299,846 | 6.19G | 0.03 | 97.93 | 94.23 | 54.40 |
| T5d-2 | 42,041,946 | 41,379,138 | 6.21G | 0.03 | 97.87 | 94.06 | 54.10 |
| T5d-3 | 40,754,908 | 40,175,196 | 6.03G | 0.02 | 97.99 | 94.39 | 54.31 |
